# Editorial: The mechanism of trace elements on regulating immunity in prevention and control of human and animal diseases

**DOI:** 10.3389/fimmu.2023.1159289

**Published:** 2023-02-23

**Authors:** Xintong Zhang, Lihua Xu, Pinnan Liu, Wenxue Ma, Yue Liu, Senqiu Qiao, Qiaohan Liu, Jingzeng Cai, Ziwei Zhang

**Affiliations:** ^1^ College of Veterinary Medicine, Northeast Agricultural University, Harbin, China; ^2^ Key Laboratory of the Provincial Education, Department of Heilongjiang for Common Animal Disease Prevention and Treatment, Harbin, China

**Keywords:** selenoprotein M(SelM), TXNRD3, NiCl2, melatonin, mice

Nickel is a hard, malleable, silver-white transition metal that is widely used in metallurgical processes such as alloy manufacturing, nickel cadmium battery manufacturing, and food industries (1). Nickel can be enriched in humans and animals through the food chain and direct contact, and when it accumulates to a certain level in the body without proper treatment, it can be harmful to living organisms as a toxic metal ion (2–4). Human exposure to excessive nickel can cause toxic side effects such as allergies, cardiovascular disease, kidney disease, pulmonary fibrosis, and cancers (5). It has been shown that nickel nanoparticles can cause reproductive toxicity in mice (6). Melatonin (MT) is an indole hormone secreted primarily by the pineal gland in mammals and humans (7). It is used for the treatment of hypertension, hyperlipidemia, myocardial injury, and myocardial ischemia reperfusion injury (8). Previous experiments by Cai et al. showed that MT ameliorates trimethyltin chloride-induced cardiotoxicity through the nuclear xenobiotic metabolism and Keap1-Nrf2/ARE axis-mediated pyroptosis.

Selenium is an essential trace element for the human body, which exists in selenoproteins in the form of selenocysteine ​​and selenomethionine, and exerts its biological functions through 25 selenoproteins. Selenoprotein M (SelM) is one of the executive factors of selenium *in vivo* and may be involved in antioxidant, neuroprotection and the regulation of intracellular calcium (9). Existing studies have shown that the overexpression of human SelM in rats increases the activity of antioxidant enzymes such as glutathione peroxidase (GPX) and superoxide dismutase (SOD) (10). In addition, SelM has a neuroprotective function in the regulation of cytosolic calcium, which plays an important role in the pathogenesis of neurodegenerative diseases (11). Of note, previous studies have shown that SelM^-/-^ mice are observed to be obese and leptin-resistant (12, 13). Our previous research showed that in a high-fat diet (HFD) induced non-alcoholic fatty liver disease (NAFLD) model, there is a significant downregulation of SelM expression. Notably, SelM contains a redox-active CXXU motif that has been shown to bind Zn^2+^ and Cu^+^, thereby acting as a metal regulator (14, 15). Thioredoxin/glutathione reductase (TXNRD3) is a selenoprotein composed of thioredoxin reductase and glutaredoxin domains. It has been shown that TXNRD3 plays an important role in male reproduction by supporting redox homeostasis during spermatogenesis (16). To investigate the role of SelM and Txnrd3 in nickel poisoning, we established a nickel exposure model by 21 days of NiCl_2_ gavage in wild-type C57BL/6N (WT) mice, SelM^-/-^ C57BL/6N (SelM^-/-^) mice, and Txnrd3^-/-^ C57BL/6N (Txnrd3^-/-^) mice.

NiCl_2_ damages the immune system, including the spleen and primary splenic lymphocytes. The splenic white and red pulp of SelM ^-/-^ mice has shown greater destruction than WT mice after nickel exposure. Ma et al. also demonstrated that oxidative stress, inflammation, and apoptosis occurred in the spleen of mice during this period. In our ongoing study of the toxicity of NiCl_2_ on primary splenic lymphocytes, we found that cells undergo oxidative stress-induced inflammation and necroptosis with increasing concentrations of NiCl_2_. When N-Acetyl-L-cysteine (NAC) alleviated oxidative stress, the inflammation and necroptosis were also reduced in the primary splenic lymphocytes (nonpublic data).

We then investigated the circulatory system by examining the heart. In Txnrd3^-/-^ mice, we found that compared with wild-type mice, reduced Txnrd3 expression promoted nickel-induced mitochondrial apoptosis and an oxidative stress-induced inflammatory response, which exacerbated cardiac injury. Specific manifestations include increased messenger mRNA levels of mitochondrial apoptosis (caspase-3, caspase-9, cytochrome c, p53, and BAX), autophagy (LC3, ATG 1, ATG 7, and Beclin-1), and inflammation (TNF-α, COX 2, IL-1, IL-2, IL-6, and IL-7), but decreased levels of bcl-2, p62, and mTOR (Liu Yue et al.). We also discovered that NiCl_2_ caused changes in the microstructure and ultrastructure of the hearts of WT and SelM^-/-^ mice, which were caused by oxidative stress, endoplasmic reticulum (ER) stress, and apoptosis, as evidenced by a decrease in the MDA content and T-AOC activity. At the same time, ER stress-related genes (ATF4, IRE-1, JNK, and CHOP) and apoptosis-related genes (Bax, Caspase-3, Caspase-9, Caspase-12, and bcl-2) changed their mRNA and protein expression. It is worth noting that SelM^-/-^ mice were more severely injured (nonpublic data).

Regarding the metabolic system, we observed changes in the liver and kidneys. In the liver, liver fibrosis was more severe, and organelle damage was more pronounced in Txnrd3^-/-^ mice compared to wild-type mice after nickel exposure. In this process, activation of the IRE1/Nuclear factor kappa B/NLRP3 16 axis leads to liver pyroptosis, while upregulation of PERK/TGF-β17 promotes the liver fibrosis process. Moreover, Txnrd3 knockdown has been found to exacerbate liver injury during nickel exposure (Liu Qi et al.). Our current findings suggest that SelM^-/-^ mice experience more severe ferroptosis caused by lipid peroxidation in the liver (nonpublic). We also confirmed that Txnrd3 knockdown can aggravate the oxidative stress and mitochondrial apoptosis caused by the increase of reactive oxygen species in the kidney caused by NiCl_2_ (nonpublic).

We also investigated the NiCl_2_ toxicology in nervous system. Neuronal brain atrophy and other neurotoxic features in brain tissue were observed in WT mice, and Qiao’s study also proposed that nickel induces oxidative stress damage and autophagy in the mouse brain through the inhibition of the PI3K/AKT/mTOR pathway.

In addition, for the respiratory system, we focused on the changes in the lung tissue after NiCl_2_ exposure. In the lung tissue of SelM^-/-^mice and WT mice, light microscopy revealed inflammatory cell infiltration, alveolar collapse, and alveolar wall thickening, while electron microscopy of the lung tissue showed a large accumulation of fibroblasts, the proliferation of collagen fibers, and dense collagen deposition. Lung fibrosis was more severe in SelM^-/-^ mice than that in WT mice. We suggest that SelM knockdown leads to epithelial mesenchymal transition through the activation of the oxidative stress-mediated TGF-β1/Smad signaling pathway and promotes lung fibrosis development (nonpublic data). In the lung tissue of Txnrd3^-/-^, pathological results showed that after exposure to nickel, the lung tissue was significantly damaged and infiltrated with inflammatory cells. Inflammatory cells and protein fragments were found in the bronchioles of Txnrd3^-/-^ mice. Ultrastructural examination showed that mitochondria in the Txnrd3^-/-^ mice were abnormal, and pulmonary capillary endothelial cells were dense and short. qPCR and WB results suggested that NiCl_2_ activated the TNF-α/NF-κB pathway by increasing the expression of HO-1, IL-1β, iNOS, and COX2 and inducing the inflammatory response (nonpublic data).

We added melatonin to the above model for intervention and found that it could significantly alleviate the damage caused by Ni poisoning in WT and knockout mice. However, in the tissues and organs of the gene knockout mice, the antagonism of melatonin on Ni was not as obvious as that of the wild-type mice, and a certain degree of damage still existed, indicating that SelM and Txnrd3 play an important role in the process of multiple tissues and organs damage caused by exposure to Ni.

All our data suggest that SelM and Txnrd3 play an important role in nickel toxicity. MT showed a protective effect in WT mice after NiCl_2_ exposure, but this effect was markedly attenuated in knockout mice. During this process, biological processes such as oxidative stress, inflammation, apoptosis, and ER stress occur. Next, we will examine the specific biological effects of SelM and Txnrd3.

## Author contributions

XZ and LX: Writing – original draft. PL and WM: Writing – review and editing. YL and SQ: Investigation, Visualization. QL and JC: References collection. ZZ: Supervision, Writing – review & editing, Funding acquisition. All authors contributed to the article and approved the submitted version.
